# Customized Reference Ranges for Laboratory Values Decrease False Positive Alerts in Intensive Care Unit Patients

**DOI:** 10.1371/journal.pone.0107930

**Published:** 2014-09-18

**Authors:** Oguz Kilickaya, Christopher Schmickl, Adil Ahmed, Juan Pulido, James Onigkeit, Kianoush Kashani, Ognjen Gajic, Vitaly Herasevich, Brian Pickering

**Affiliations:** 1 Multidisciplinary Epidemiology and Translational Research in intensive care, emergency and perioperative Medicine (METRIC), Division of Pulmonary and Critical Care Medicine, Department of Medicine, Mayo Clinic, Rochester, Minnesota, United States of America; 2 University Witten-Herdecke, Witten, Germany; 3 Division of Critical Care, Department of Anesthesiology, Mayo Clinic, Rochester, Minnesota, United States of America; 4 Division of Pulmonary and Critical Care, Department of Medicine, Mayo Clinic, Rochester, Minnesota, United States of America; 5 Division of Nephrology and Hypertension, Department of Medicine, Mayo Clinic, Rochester, Minnesota, United States of America; Oregon Health and Science University, United States of America

## Abstract

**Background:**

Traditional electronic medical record (EMR) interfaces mark laboratory tests as abnormal based on standard reference ranges derived from healthy, middle-aged adults. This yields many false positive alerts with subsequent alert-fatigue when applied to complex populations like hospitalized, critically ill patients. Novel EMR interfaces using adjusted reference ranges customized for specific patient populations may ameliorate this problem.

**Objective:**

To compare accuracy of abnormal laboratory value indicators in a novel vs traditional EMR interface.

**Methods:**

Laboratory data from intensive care unit (ICU) patients consecutively admitted during a two-day period were recorded. For each patient, available laboratory results and the problem list were sent to two mutually blinded critical care experts, who marked the values about which they would like to be alerted. All disagreements were resolved by an independent super-reviewer. Based on this gold standard, we calculated and compared the sensitivity, specificity, positive and negative predictive values (PPV, NPV) of customized vs traditional abnormal value indicators.

**Results:**

Thirty seven patients with a total of 1341 laboratory results were included. Experts’ agreement was fair (kappa = 0.39). Compared to the traditional EMR, custom abnormal laboratory value indicators had similar sensitivity (77% vs 85%, P = 0.22) and NPV (97.1% vs 98.6%, P = 0.06) but higher specificity (79% vs 61%, P<0.001) and PPV (28% vs 11%, P<0.001).

**Conclusions:**

Reference ranges for laboratory values customized for an ICU population decrease false positive alerts. Disagreement among clinicians about which laboratory values should be indicated as abnormal limits the development of customized reference ranges.

## Introduction

Audiovisual notifications have widely been used in patient care areas to provide information about organ and device function in order to attract health-care providers’ attention to an abnormality for a possible immediate action.

Early in the 1970s, laboratories started actively notifying health care providers about “critical” or “panic” values, indicating potential life-threatening conditions. [1] Since then, clinical laboratories have been required to list the normal ranges and develop notification procedures to alert clinicians [2].

Early detection and response to abnormal laboratory values is crucial in critical care. [3] However, there are two main barriers that hinder early detection of abnormalities in critically ill patients. Large quantities of available data can lead to information overload, which potentially limits the quality of decisions in critical care settings. [4] Furthermore, it has been shown that some commonly presented data points have no meaningful clinical impact on the care of the critically ill patients. [5] On the other hand normal ranges listed by clinical laboratories and traditional electronic medical (EMR) systems are often defined based on blood analyses of healthy men and non-pregnant women aged between 20–50 years. [6] However, these values may be normal or at least acceptable in specific populations like elderly patients or an ICU setting. [6], [7] For example, a hemoglobin of 9.5 mg/dl may raise concerns at the family physician’s visit in a healthy person but be fully acceptable in non-bleeding patients in the ICU. Thus, when applied across the various hospital populations, such a traditional, “one size fits all” approach is therefore prone to increase false positive alerts, which can dangerously desensitize health care providers and thus prevent recognition of true abnormalities [8]–[10].

Therefore we hypothesized that an EMR system selecting and marking laboratory values as abnormal based on patients’ specific situation should reduce these barriers.

While the “traditional” EMR interface at our institution indiscriminately displays all available laboratory values alongside standard reference ranges, we recently implemented a new EMR interface tailored towards ICU provider needs thereby improving their performance (lower NASA-taskload index, faster extraction of key information with less errors). [11] One important feature of this “novel” EMR interface is that laboratory data is selected and flagged as abnormal based on surveys of critical care experts about what is meaningful in an ICU setting [12].

The primary objective of this study was to compare sensitivity, specificity and predictive values for abnormal laboratory value indicators between two EMR systems (novel versus traditional) using as reference standard the judgment of board certified critical care physicians about which data they would like to be alerted given patients’ particular clinical situations.

## Methods

The study was conducted at a tertiary care academic medical center. We compared two EMR interfaces (“traditional” [13] versus “novel” [14]) currently in use at our institution with regards to the accuracy of abnormal value indicators (“alerts”) of laboratory findings. Over a two-day period we enrolled all patients consecutively admitted to any of four ICUs (medical, surgical, mixed and trauma). Patients younger than 18 years of age, those who denied research authorization, and readmissions or those who transferred between ICUs were excluded.

For each eligible patient we simultaneously took screenshots of the two EMR interfaces’ laboratory sections at a random time point during the day of admission. Based on the screenshots a list of all available laboratory tests was created omitting abnormal value indicators of either EMR interface (for each available laboratory test only the most current value was chosen, i.e. the list did not contain any information about temporal trends). This list together with each patient’s problem list was sent to two board certified critical care physicians who independently marked the test results, about which they would like to be alerted given the patient’s diagnoses (“gold standard”). Interobserver agreement between physicians was assessed using Cohen’s Kappa. Disagreements were resolved by a third board certified critical care physician making the final decision.

Patient demographics including age, gender, admission diagnosis, severity of illness (APACHE III), ICU/hospital length of stay and mortality along with mechanical ventilation status were queried from a previously validated ICU database (Multidisciplinary Epidemiology and Translational Research in Intensive Care [METRIC] ICU Datamart) [15].

Descriptive patient data were summarized as median and inter-quartile ranges (IQR) or percent (number) as appropriate. For each EMR interface we calculated sensitivity, specificity and predictive values of abnormal value indicators as compared to the gold standard. For each measure of accuracy we determined exact binomial confidence intervals and used two-sample proportion tests to compare them across the two EMR interfaces. A p-value <0.05 was considered as statistical significant. All analyses were performed using STATA 12.1 (StataCorp, College Station, TX).

### Ethics Statement

This study was approved by the Mayo Clinic Institutional Review Board (IRB #13-003762). The IRB waived the need for informed consent as doing so would not have been feasible and the risk from this study for patients was minimal. Only patients who gave permission to use their medical record for research on admission (“research authorization”) were included into this study.

## Results

**Figure 1:**
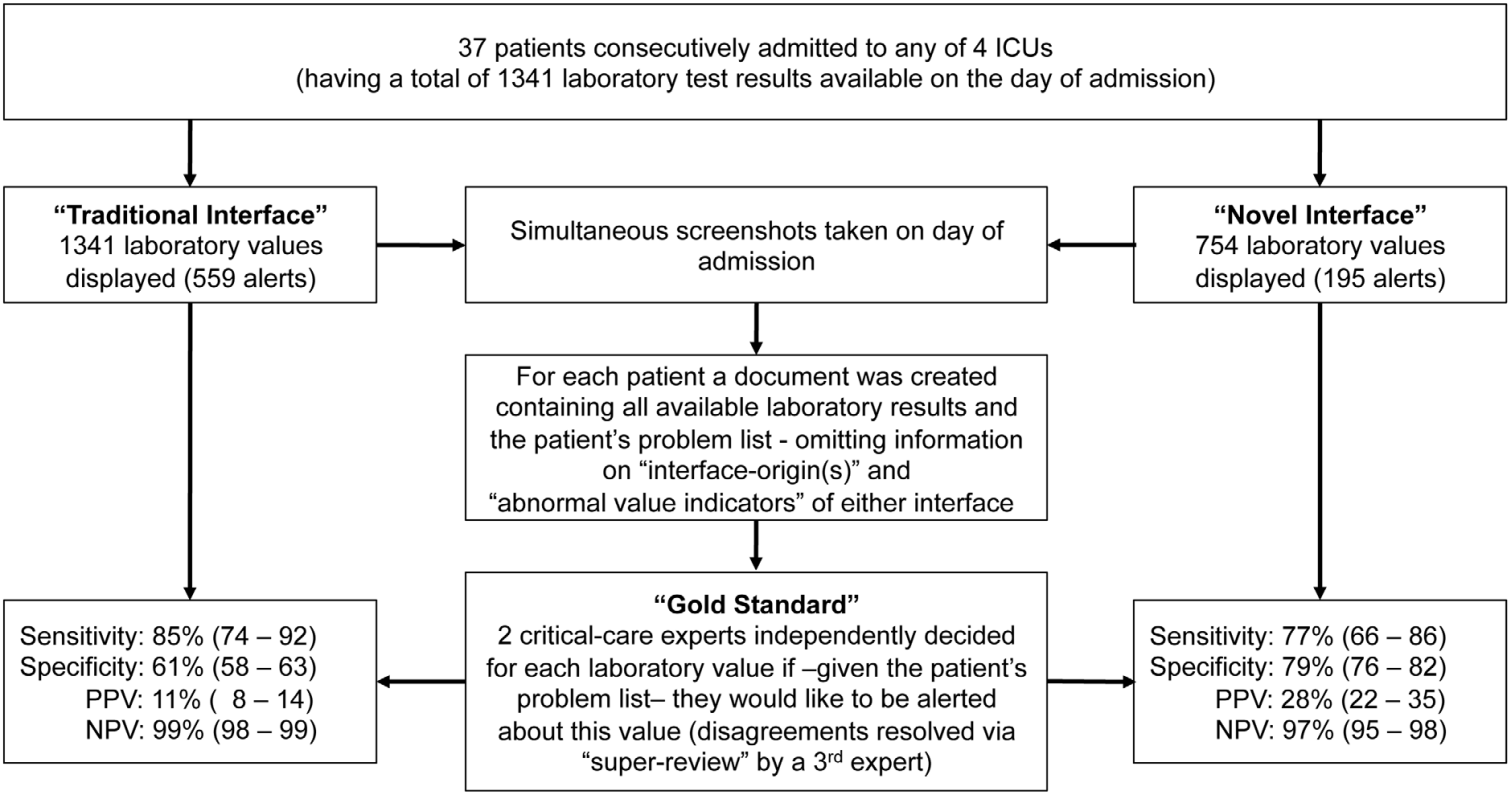
Studyflow and Results. Sensitivity, Specificity, Positive and Negative Predictive Values (PPV, NPV) are given as estimate (95%-Confidence Interval). Only specificity and negative predictive values differed significantly (for details see text).

Over the two-day period we enrolled 37 patients (Table 1). For these a total of 1341 laboratory values were reported in the traditional EMR interface of which 754 (56%) values were also displayed in the novel interface (Figure 1). The percentage of laboratory values indicated as abnormal was 42% (559/1341) in the traditional interface compared to 26% (195/754) in the novel interface.

**Table 1:** Patient Characteristics.

Patient Characteristic (n = 37)	Median (IQR) or percent (number)
Age (years)	71.4 (51.2; 84.0)
Female (%)	57 (21)
APACHE III Score	63 (43; 74)
ICU LOS (days)	1.67 (0.8; 2.8)
Hospital LOS (days)	5.8 (2.0; 7.8)
ICU Mortality (%)	2.7 (1)
Hospital Mortality (%)	13.5 (5)
Invasive ventilation (%)	24.3 (9)

Abbreviations: IQR Inter-quartile Range, LOS Length of stay, n sample size.

**Figure 2:**
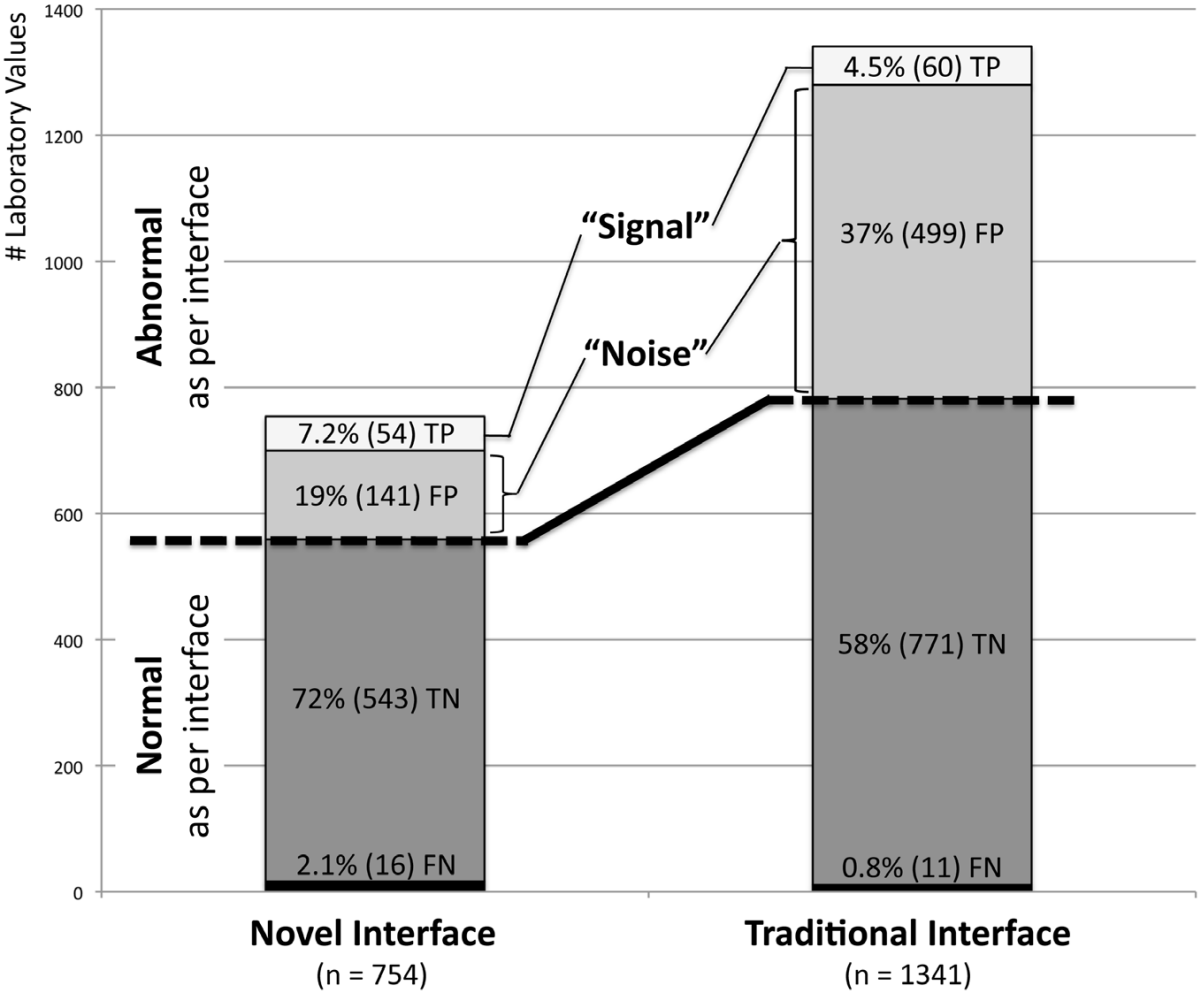
Normal and abnormal Laboratory Values displayed by both Interfaces subclassified according to Gold Standard Judgment. Percentage of true positive (TP), false positive (FP), true negative (TN) and false negative (FN) values shown relative to the total number of laboratory values displayed by each interface as percent (number). Truly abnormal laboratory test results (TP) commonly signal health-care providers the need to take action with regards to their patients’ health status. Laboratory values falsely indicated as abnormal (FP) represent in this sense a distraction or “noise” clouding this important “signal”. While an abnormal value in the traditional interface reflects a true abnormality in roughly 1 out of 9 times this “signal-to-noise ratio” is 1 in 4 (i.e. more than twice as high) in the novel interface.

Experts had fair agreement (kappa = 0.39) classifying 71 of all 1341 (5.3%) values as abnormal. One of these 71 (1.4%) “truly abnormal” values was not displayed in the novel interface (serum-Hydroxybutyrate). Figure 2 provides details on how many of the values displayed as abnormal and normal in either interface were true or false as per expert review, respectively.

Compared to the traditional interface, the novel interface had a similar sensitivity (77% versus 85%, P = 0.22) and negative predictive value (97.1% versus 98.6%, P = 0.06) but had a higher specificity (79% versus 61%, P<0.001) and positive predictive value (28% versus 11%, P<0.001).

## Discussion

The novel interface selectively omitted almost half of all available laboratory data and among all displayed values marked 38% less values as abnormal compared to the traditional interface. The main effect was a substantial reduction in the number of values indicated as abnormal by the interface but considered normal by critical care experts (false positive alerts), which was reflected by the higher specificity and positive predictive value of the novel interface. Of note, this was achieved without substantially increasing the number of values indicated as normal by the interface but considered abnormal by ICU physicians (false negatives), thus maintaining the same sensitivity and negative predictive value as the traditional interface.

Truly abnormal laboratory test results (true positives) commonly signal health-care providers the need to take action. Laboratory values falsely indicated as abnormal (false positives) represent in this sense a distraction or “noise” clouding this important “signal” (Figure 2). In clinical practice bedside physicians are most interested in knowing how likely patients are to really have the disease indicated by a positive test result. This information is conveyed by the positive predictive value (PPV). While an abnormal value in the traditional interface reflects a true abnormality in roughly 1 out of 9 times (PPV = 11%) this “signal-to-noise ratio” is 1 in 4 (PPV = 28%; i.e. more than twice as high) in the novel interface. This may reduce the initially discussed alert-fatigue that providers often face, and may in part explain the improved performance of health-care providers. [11] There was a trend towards a smaller negative predictive value (NPV) in the novel interface, but whether a health-care provider can be 97.1% or 98.6% certain that a value not marked as abnormal is truly normal is clinically irrelevant.

Our finding that the standard reference ranges in the traditional interface produce a large fraction of false positive notifications (37%) is consistent with reports from other studies evaluating alerts in ICU settings more generally [8]–[10].

Strengths of our study include the consecutive inclusion of patients representing a broad spectrum of the typical ICU population (which means a representative prevalence of truly abnormal results allowing valid estimation of PPV and NPV) and that expert reviewers were blinded to abnormal value indicators as well as the underlying reference ranges in the novel EMR interface (none of the experts was part of the expert panels used to define customized reference ranges or involved in the design of the novel interface).

The major limitation is that agreement between expert reviewers was only fair. Since truly abnormal values often mean that further actions are warranted, this relative disagreement likely reflects to a large extent the inherently different thresholds different physicians have to initiate further testing or treatment based on abnormal values. We tried to ameliorate this problem by using *experienced* critical care physicians as reviewers and resolving disagreements by a super-review. But while the absence of a formally established gold standard limits the validity of our evaluation – on a more conceptual level­ it also represents a fundamental barrier for developing clinically meaningful reference ranges based on experts’ opinion or practice.

Another important limitation is that the presented measures of accuracy do not reflect that one of the test results (beta-hydroxybutyrate) judged as abnormal by the expert review was not even included in the novel interface. By precluding provider action, the consequences arising from the omission of even a few truly abnormal results may outweigh the benefits of substantially reducing the amount of low-value data and subsequent information overload. [4] One has to take into account, however, that (1) only one out of 71 (1.4%) truly abnormal results was omitted, (2) the missed test result is only of interest in very special clinical circumstances (differential diagnosis of ketoacidosis), (3) the novel interface is designed to be an efficient first place to get an overview over a patient’s health status with low-value data (which occasionally may be of great interest) remaining accessible in the underlying EMR system, (4) one would expect health-care providers to follow up such specific tests in the underlying EMR system, and (5) the overall probability that for a given patient a truly abnormal result is not displayed or not marked as such is similar in the novel vs the traditional interface (23.9% vs 15.5%, P = 0.21).

While abnormal values are what matters most for clinicians, there may be times when normal test results may be just as –or even more– important. Our study has not been designed to and can thus not provide any information about whether important “normal” results were lacking in the novel interface. Based on the novel interface’s positive impact on health-care providers’ performance, [11] it is, however, unlikely that the latter two discussed limitations constitute substantial problems in clinical practice.

Furthermore, this study was designed to test the *feasibility* of using customized reference ranges derived from expert opinion to decrease false positive alerts in an ICU setting in general. While this approach appears to be effective overall, the sample size of 37 patients (and thus a maximum of 37 values for each test) precludes meaningful subgroup analyses to compare performance between the two EMR interfaces on a per laboratory-test level. Future studies should incorporate a larger sample size (of patients and expert reviewers) to enable this and other subgroup analyses comparing performance stratified by diagnosis (GI bleed vs sepsis), different ICUs (medical vs surgical) and regional practices (multi-center study).

Lastly, it is unclear to what extent the reduction of false positive alerts by 38% will translate into a decrease in alert fatigue and if a threshold effect exists. Apart from the obvious difficulty to measure alert fatigue, we can only speculate that the relationship between these two variables is curvilinear: a small decrease in the number of false positive alerts is likely to go unnoticed by healthcare providers, whereas a medium or large reduction should be associated with a change in alert fatigue of similar magnitude. However, even a big change may have only negligible impact if the *absolute* number of false positive alerts in a given time-period is exceedingly high (e.g. a 38% reduction from 100 to 62 alerts per day is likely to have more impact than one from 10,000 to 6,200). Further studies are needed to verify these hypotheses and better characterize this relationship.

While our results are encouraging, a next step should be to develop reference ranges for laboratory values which are not only adapted to specific hospital populations (in this case ICU patients) but which adjust for more specific patient characteristics such as age, gender, medical conditions, treatments or –in the future– even genes (“patient-customized” reference ranges). Furthermore, the usage of laboratory value trends rather than absolute numbers should be considered to improve accuracy of abnormal value indicators. Apart from the obviously increased complexity of such endeavors, any approach incorporating expert opinions will, however, be limited by the relative high degree of disagreement among such experts.

## Conclusions

Reference ranges for laboratory values adjusted towards a population of critically ill patients based on expert consensus decreased false positive alerts (increased specificity and positive predictive value) without affecting the fraction of false negative alerts (unchanged sensitivity and negative predictive value). This may reduce alert fatigue among health-care providers. Substantial disagreement among critical care experts about which laboratory values should be indicated as abnormal is a major limitation for both developing and testing such customized reference ranges.

## Supporting Information

Dataset S1 (XLSX)
